# epHero – a tandem-fluorescent probe to track the fate of apoptotic cells during efferocytosis

**DOI:** 10.1038/s41420-024-01952-1

**Published:** 2024-04-17

**Authors:** Sanjna Singh, Julien Bensalem, Leanne K. Hein, Aaron Casey, Ville-Petteri Mäkinen, Timothy J. Sargeant

**Affiliations:** 1 South Australian Health and Medical Research Institute, Adelaide, SA Australia; 2 University of Adelaide, Adelaide, SA Australia; 3 Research Unit of Population Health, Faculty of Medicine, University of Oulu, Oulu, Finland; 4 Biocenter Oulu, University of Oulu, Oulu, Finland

**Keywords:** Apoptosis, Lysosomes

## Abstract

The efficient removal of apoptotic cells via efferocytosis is critical for maintaining optimal tissue function. This involves the binding and engulfment of apoptotic cells by phagocytes and the subsequent maturation of the phagosome, culminating in lysosomal fusion and cargo destruction. However, current approaches to measure efferocytosis rely on labelling apoptotic targets with fluorescent dyes, which do not sufficiently distinguish between changes to the engulfment and acidification of apoptotic material. To address this limitation, we have developed a genetically coded ratiometric probe epHero which when expressed in the cytoplasm of target cells, bypasses the need for additional labelling steps. We demonstrate that epHero is a pH-sensitive reporter for efferocytosis and can be used to simultaneously track changes to apoptotic cell uptake and acidification, both in vitro and in mice. As proof-of-principle, we modify extracellular nutrition to show how epHero can distinguish between changes to cargo engulfment and acidification. Thus, tracking efferocytosis with epHero is a simple, cost-effective improvement on conventional techniques.

## Introduction

The human body turns over nearly four million cells per second [1]. These cells are recognised and cleared in a process termed efferocytosis – derived from the Latin *effere*, which means ‘to carry to the grave’. Under physiological conditions, apoptotic cells are rapidly removed by phagocytes – both professional and non-professional – resulting in a largely anti-inflammatory response and the maintenance of homeostasis [2]. When this process is hampered, apoptotic cells undergo secondary necrosis, releasing intracellular material that causes inflammation and tissue necrosis [3–5]. Perturbations in efferocytosis have been linked to several disease processes, including autoimmunity [6, 7], atherosclerosis [8], and the tumour microenvironment [9].

At present, two techniques are conventionally used to measure efferocytosis: (i) flow cytometric or (ii) microscopic analysis of phagocytes ingesting labelled targets [10, 11]. Flow cytometry enables the analysis of hundreds of thousands of cells in a relatively short period of time; it does not, however, permit discrimination between material that has merely bound to the surface of phagocytes from that which has been internalised. Whilst microscopic examination allows this distinction to be made, it can be challenging to perform in a high throughput manner and may be inaccessible to some laboratories due to the high cost, time and complexity involved. Several groups have attempted to circumvent this issue by labelling targets with pH-sensitive dyes such as pHrodo [10, 11] and CypHer5E [5]. These dyes exhibit minimal fluorescence in high pH environments. Upon encountering low pH, such as within a phagosome, they fluoresce brightly: this ensures that surface-bound targets are excluded from all analyses. While this can be beneficial in some circumstances, the binding and recognition of apoptotic cells by phagocytes is a crucial first step in efferocytosis, and this is not measurable using pH-sensitive dyes. Further, since pHrodo has a single pKa [12] the degradation of apoptotic material cannot be tracked using this method, as an increase in pHrodo signal could indicate either greater uptake or acidification of cargo by phagocytes.

To address these gaps, we have adapted a concept employed in the field of autophagy [13], which studies the degradation of intracellular material. We have constructed a novel tandem-fluorescent probe called ‘epHero’, which comprises an mCherry-eGFP fusion protein to discriminate between efferocytic uptake and degradation. eGFP has a pKa of 6 [14, 15], causing its fluorescence to quench in mildly acidic environments such as those found within a late endosome or phagosome. In contrast, mCherry, with a lower pKa of 4.5 [16], is resistant to lysosomal proteases [17], enabling it to continue fluorescing even within the acidic lysosome. By expressing this tandem probe in apoptotic cells, these differences between eGFP and mCherry fluorophores can be used to track the internalisation and degradation of apoptotic cargo within phagocytes. We show that epHero is a simple, cost-effective probe that can be used to monitor the dynamics of apoptotic corpse clearance both in vitro and in mice.

## Results

### Development of mCherry-eGFP ‘epHero’ to measure efferocytic flux

**Fig. 1:**
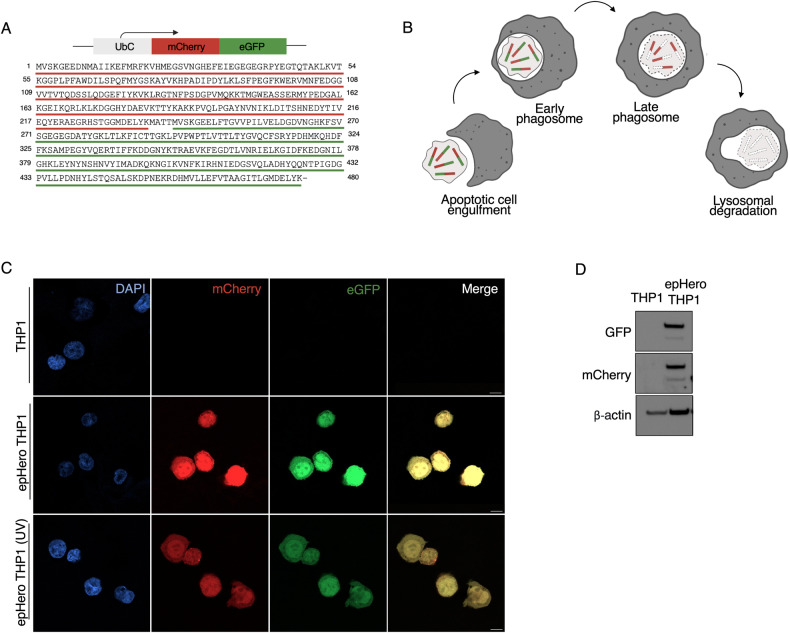
Designing a tandem-fluorescent reporter to measure efferocytic flux. **A** Amino acid sequence of the epHero reporter. Red, green: sequence encoding the mCherry and eGFP proteins, respectively, downstream of the ubiquitin C promoter. **B** Schematic of epHero probe. When expressed in apoptotic cells, changes in red and green fluorescence can be used to measure trafficking along the phagosome-lysosome pathway. **C** Confocal imaging of wild-type, live or apoptotic epHero-transduced THP1 cells. Scale bar 10 µm. **D** Immunoblots of apoptotic wild-type and epHero-transduced THP1 cell lysates probed for mCherry and eGFP.

The lumen of an apoptotic corpse-containing phagosome becomes more acidic as it matures along the phagosome-lysosome pathway [18, 19]. To engineer a probe capable of measuring efferocytosis flux, we stably transduced THP1 or Jurkat cells with a tandem fluorescent mCherry-eGFP reporter, which we termed epHero (Fig. 1A). When cells expressing the epHero probe are rendered apoptotic and co-cultured with phagocytes, eGFP should photo-quench in acidic phagosomes [15], with mCherry fluorescence remaining stable [16, 17]. Therefore, the ratio of mCherry to eGFP fluorescence in the phagocyte should increase over time, enabling the dissemination of corpse digestion and acidification (Fig. 1B). We verified expression of our tandem-fluorescent reporter in both live and apoptotic cells (Fig. 1C, D).

### Expression of epHero in apoptotic cells enables the quantification of efferocytosis

**Fig. 2:**
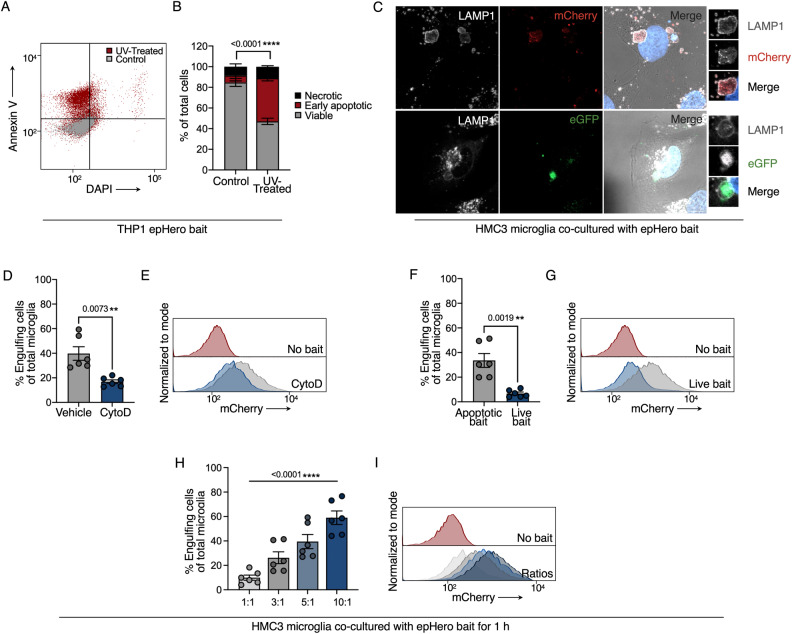
The epHero reporter can be used for the quantification of efferocytosis. **A** Representative scatter plots from flow cytometry-based live-dead staining of epHero THP1 cells following UV-induced apoptosis. **B** Percentage of viable (Annexin V− DAPI−), early apoptotic (Annexin V+ DAPI−), and necrotic (Annexin V+ DAPI+ or Annexin V− DAPI+) cells from **A**. Values represent the mean ± SEM from *n* = 5–6 across two independent experiments (two-way ANOVA with Šídák’s multiple comparisons test; *p* value on graph represents the comparison of early apoptotic cells between the control and UV-treated conditions). **C** Representative images of HMC3 microglia co-cultured with apoptotic THP1 epHero bait for 2 h and immunostained for LAMP1, eGFP and mCherry. **D** Quantification of the percentage of phagocytosing cells in microglia treated with vehicle or 10 µM cytochalasin D (CytoD) for 10 min before being co-cultured with apoptotic epHero bait for 1 h. **E** Representative histograms from **D**. Grey: Vehicle; Blue: CytoD. **F** Percentage of phagocytosing microglia upon co-culture with either live or UV-treated epHero bait for 1 h before analysis via flow cytometry. **G** Representative histograms from **E**. Grey: apoptotic bait; Blue: live bait. **H** Microglia incubated with increasing bait:phagocyte ratios (left to right) of epHero bait for 1 h. **I** Representative histograms from **H**. Graded shades of grey to blue represent ratios displayed in **H**. **D**, **F**, **H** Data represent mean ± SEM, with *n* = 6 from three independent experiments. *P* values are derived from either a t-test (**D**, **F**), or one-way ANOVA with a test for linear left-to-right p-trend (**H**).

We used UV radiation to induce apoptosis [20] in cells stably transduced with the epHero reporter. This generated an Annexin V + DAPI- early apoptotic cell population (Fig. 2A, B). The entire ‘bait’ cell mixture comprising viable, early apoptotic and necrotic cells was utilised in all subsequent efferocytosis experiments. In a physiological context, it is unlikely for phagocytes to be surrounded solely by apoptotic cells [21]. Therefore, exposing phagocytes in culture to a heterogenous mixture containing both putative efferocytosis targets and non-apoptotic cells provides a better representation of apoptotic cell clearance in an organism. We used immunocytochemistry to confirm phagolysosomal delivery of apoptotic THP1 or Jurkat epHero bait in HMC3 microglia and mouse bone marrow-derived macrophages respectively (Fig. 2C, Supplementary Fig. 1A). For analysis of efferocytosis using epHero and flow cytometry, we used the gating strategy displayed in Supplementary Fig. 2. To verify our probe’s specificity, we pre-treated microglia with cytochalasin D, an actin polymerisation inhibitor which prevents the uptake of apoptotic cells [22], before performing the efferocytosis assay. As expected, cytochalasin D reduced the uptake of epHero bait (Fig. 2D, E). To ensure that our assay was capturing true efferocytosis events and not the entotic cannibalism of live cells [23] we co-cultured both live and UV-irradiated epHero bait cells separately with microglia and measured microglial fluorescence. The uptake of epHero bait was markedly reduced in microglia co-cultured with live cells, suggesting little contribution of live cell uptake to our efferocytosis measurements (Fig. 2F, G). Previous work has shown that the binding and engulfment of apoptotic cells by phagocytes is dependent on the ratio of target cells to phagocytes [24]. We tested our probe’s ability to detect these changes in apoptotic cell uptake by co-culturing microglia with varying ratios of bait:phagocyte. In line with prior studies, our results showed that the uptake of apoptotic corpses increased when the ratio of corpses to phagocytes was higher (Fig. 2H, I). Together, these data show that our epHero-based probe can be utilised to detect changes in the uptake of apoptotic cells.

### An epHero-based approach can be used to detect pH changes during efferocytosis

**Fig. 3:**
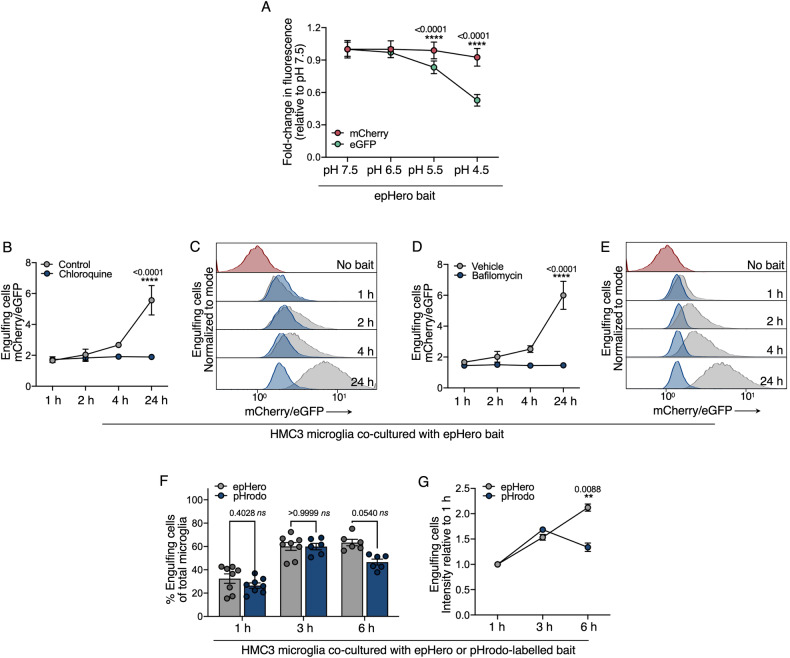
epHero is a pH-sensitive reporter of phagosomal acidification during efferocytosis. **A** Relative mCherry and eGFP signal from epHero THP1 cells incubated in different pH buffers. Data represent mean ± SEM, *n* = 4 from two independent experiments (two-way ANOVA with Šídák’s multiple comparisons test). **B** mCherry/eGFP ratio from microglia that have phagocytosed cargo after being co-cultured with apoptotic epHero bait for 1-, 2-, 4- or 24 h, with or without 50 µM chloroquine. Data represent mean ± SEM from *n* = 3 independent experiments (two-way ANOVA with Šídák’s multiple comparisons test). **C** Representative histograms from **B**. Grey: Control; Blue: Chloroquine. **D** mCherry/eGFP ratio from microglia that have phagocytosed cargo after being co-cultured with apoptotic epHero bait for 1-, 2-, 4- or 24 h, with or without 100 nM bafilomycin. Values denote mean ± SEM from *n* = 3 independent experiments (two-way ANOVA with Šídák’s multiple comparisons test for a difference between treatment groups at each time-point). **E** Representative histograms from **D**. Grey: Vehicle; Blue: Bafilomycin. **F** Percentage of phagocytosing cells from microglia co-cultured with either epHero or pHrodo-labelled apoptotic THP1 bait for 1-, 3- or 6 h. Data points centred on mean ± SEM; *n* = 6–8 from three- to four biologically independent experiments (two-way ANOVA with Šídák’s multiple comparisons test). **G** epHero (mCherry/eGFP) or pHrodo signal intensity of phagocytosing microglia from **F**. Data represent mean ± SEM from *n* = 6–8 across three- to four independent experiments (two-way ANOVA with Šídák’s multiple comparisons test).

We next determined whether the epHero reporter could be employed to measure changes to the acidification and degradation of apoptotic corpses by phagocytes. First, we tested changes to the fluorescence of epHero bait cells in response to varying pH. Upon a reduction in pH, mCherry fluorescence remained stable whereas eGFP signal decreased (Fig. 3A), highlighting the pH-responsive nature of our probe.

As phagosomes mature and fuse with lysosomes, their luminal pH decreases [18, 19]. We modulated this process by treating phagocytes with chloroquine and bafilomycin, two inhibitors of phagosomal acidification that act via distinct mechanisms [25, 26]. When chloroquine or bafilomycin-treated microglia were co-cultured with epHero bait, their corpse-containing phagosomes failed to acidify, as evidenced by the complete lack of mCherry/eGFP signal development over time (Fig. 3B–E). This occurred despite only minor changes to the uptake of apoptotic corpses by microglia (Supplementary Fig. 3A, B). Next, we compared epHero to pHrodo, another pH-sensitive reporter that is commonly used to track efferocytosis [11]. pHrodo-labelled targets exhibit weak signal in neutral environments, with their fluorescence increasing in a pH-dependent manner following cargo internalisation. We co-cultured microglia with epHero-expressing or pHrodo-labelled apoptotic bait and tracked fluorescent signal development within microglia over time. At early time-points (<3 h), signal development was comparable between the two probes. However, there was a trend towards less uptake detected in microglia cultured with pHrodo-labelled bait at 6 h (Fig. 3F, *p* = 0.0540). Further, at 6 h post-treatment there was a sizeable decrease in phagosome acidification as detected by pHrodo compared to epHero, likely due to the complete digestion of a subset of pHrodo-labelled cargo by phagocytes (Fig. 3G). In line with this, eGFP fluorescence was lowest 6 h post-treatment (Supplementary Fig. 3C), indicating that complete quenching of the pHrodo probe affects its ability to detect efferocytosis flux at its terminal stages. Thus, the epHero reporter is pH-sensitive and can detect changes to phagosomal acidification in phagocytes.

### The epHero reporter can track changes to efferocytosis over time

**Fig. 4:**
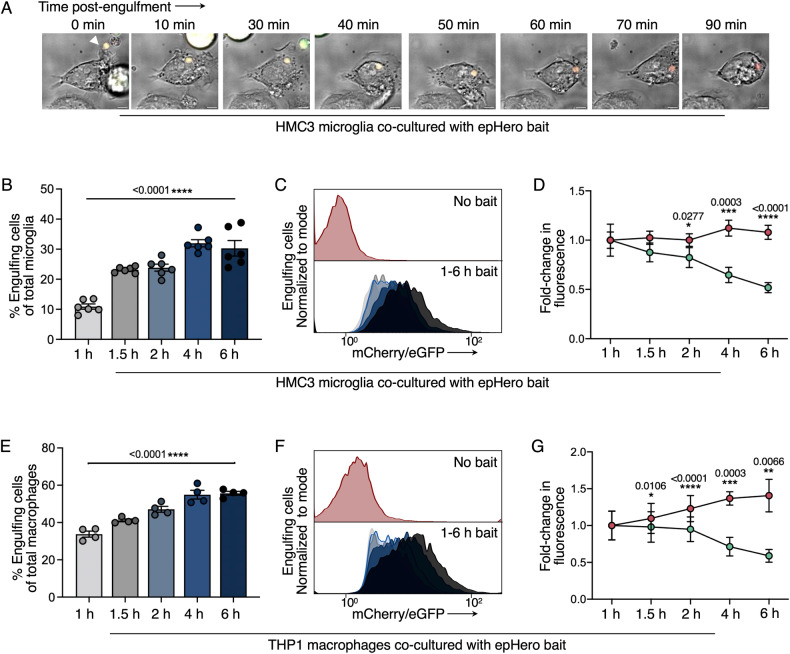
epHero can track changes in efferocytic flux over time. **A** Stills taken from confocal live imaging of microglia co-cultured with apoptotic epHero bait for 2 h. **B** Percentage of phagocytosing cells from microglia co-cultured with apoptotic epHero bait for up to 6 h. Data points centred on mean ± SEM with *n* = 6 from three independent experiments (one-way ANOVA with test for linear left-to-right trend). **C** Representative flow cytometry histograms from **B**. **D** Fold-change in fluorescent signal (relative to 1 h) over time in phagocytosing microglia from **B**. Red: mCherry fluorescence; Green: eGFP fluorescence. Data points denote mean ± SD (two-way ANOVA with Šídák’s multiple comparisons test). **E** Percentage of phagocytosing cells from THP1 macrophages co-cultured with apoptotic epHero bait for up to 6 h. Data points centred on mean ± SEM with *n* = 4 from two independent experiments (one-way ANOVA with test for linear left-to-right p-trend). **F** Representative flow cytometry histograms from **E**. **G** Fold-change in fluorescence signal (relative to 1 h) over time in phagocytosing THP1 macrophages from **E**. Red: mCherry fluorescence; Green: eGFP fluorescence. Data points denote mean ± SD (two-way ANOVA with Šídák’s multiple comparisons test).

To determine whether our probe would be useful for tracking temporal changes in efferocytosis, we co-cultured microglia with apoptotic epHero bait for varying lengths of time. We were able to detect the acidification of engulfed apoptotic cargo via live cell microscopy (Fig. 4A, Supplementary Video 1). Flow cytometry revealed that the proportion of efferocytic microglia increased over time (Fig. 4B); examination of cell fluorescence showed an increase in the ratio of mCherry to eGFP signal – the readout of phagosomal acidification (Fig. 4C). While mCherry fluorescence remained relatively stable, eGFP fluorescence decreased progressively as the corpse-containing phagosome matured and became more acidic over time (Fig. 4D). Given that all our previous measurements were performed in human microglia, it was important to ensure that our probe was also capable of tracking efferocytosis in macrophages. Consistent with our microglia data, incubating THP1 or bone marrow-derived macrophages with epHero bait yielded both increased uptake and acidification of the apoptotic corpse-containing phagosome over time (Fig. 4E–G, Supplementary Fig. 1B–D). Taken together, these data demonstrate that epHero can be used to measure temporal changes in both cargo uptake and acidification during efferocytosis in these different cell types.

### The epHero probe can be used for the ex vivo measurement of efferocytic flux

**Fig. 5:**
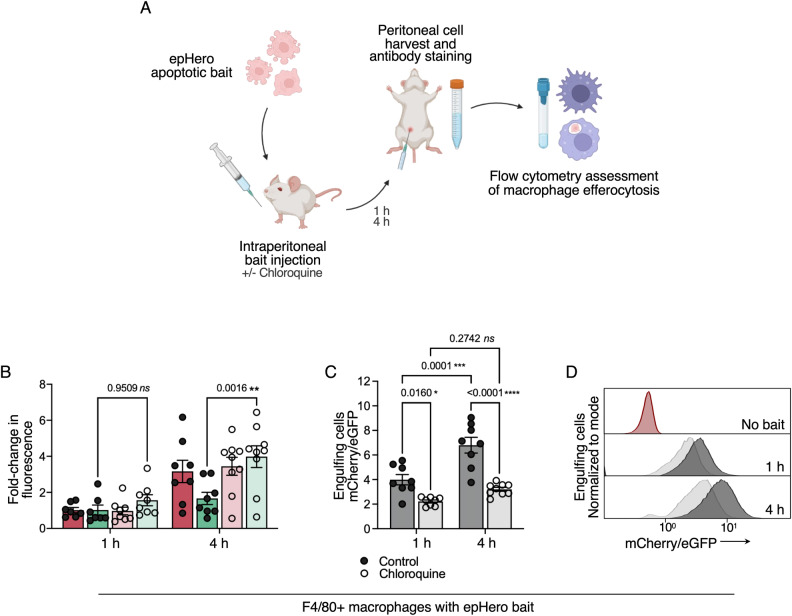
The epHero reporter is a pH-responsive measure of efferocytosis in mice. **A** Schematic showing the experimental design to measure efferocytic flux in control or chloroquine-treated mice. **B** Fold-change in fluorescence (relative to 1 h) in F4/80+ peritoneal macrophages from control (closed circles) or chloroquine-treated (open circles) mice 1- to 4 h following injection with apoptotic epHero bait. Red: mCherry fluorescence; Green: eGFP fluorescence. Data points are centred on mean ± SEM, with 7–9 mice per condition (two-way ANOVA with Šídák’s multiple comparisons test). **C** mCherry/eGFP ratio of phagocytosing F4/80+ peritoneal macrophages from **B**. Error bars represent SEM, *n* = 7–9 mice per condition (two-way ANOVA with Šídák’s multiple comparisons test). **D** Representative flow cytometry histograms denoting mCherry/eGFP from **C**. Black: Control; Grey: Chloroquine. ns not significant.

We sought to ascertain whether the epHero reporter could be used to provide information on efferocytosis in organisms. The peritoneal cavity of mice contains a sizeable macrophage population, and the injection of material into the mouse peritoneal cavity is a well-established method to study in vivo phagocytosis by proxy [27, 28]. We injected apoptotic epHero bait into the peritoneal cavity of mice and harvested the peritoneal exudate at 1- and 4 h time-points. To further ensure that our probe provided a pH-sensitive measurement of efferocytosis, mice were also injected with either saline or the lysosomotropic agent chloroquine at the same time (Fig. 5A). Akin to our in vitro measurements, the mCherry/eGFP ratio was higher at 4 h compared to 1 h post-injection, consistent with an increase in phagosomal acidification over time (Fig. 5C, D, gating strategy shown in Supplementary Fig. 4A). Chloroquine treatment abolished this response, with the macrophages from chloroquine-injected mice having consistently reduced mCherry/eGFP ratios compared to their saline-injected counterparts. Further, this ratiometric response was primarily driven by changes to eGFP fluorescence, with mCherry fluorescence and cargo uptake remaining consistent between the saline and chloroquine treatment groups (Fig. 5B, Supplementary Fig. 4B). Overall, these results demonstrate that epHero can be used to simultaneously evaluate both cargo uptake and acidification during efferocytosis in mice.

### Serum and amino acid availability respectively modulate the uptake and acidification of apoptotic material during efferocytosis

**Fig. 6:**
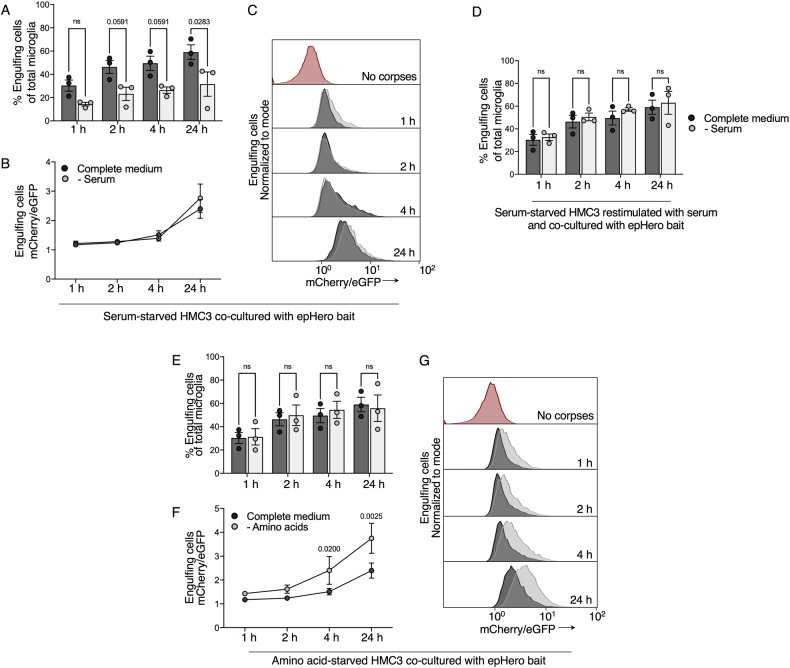
The epHero reporter reveals nutrition-mediated changes to corpse uptake and acidification during microglial efferocytosis. **A** Quantification of the percentage of phagocytosing cells from serum-fed or -starved microglia co-cultured with epHero bait for up to 24 h. Dark grey: complete medium; light grey: without serum. **B** mCherry/eGFP ratio from microglia that have phagocytosed cargo after being co-cultured with apoptotic epHero bait for up to 24 h, with or without serum starvation. **C** Representative histograms from **B**. **D** Quantification of the percentage of phagocytosing cells from serum-fed or -starved (and re-stimulated) microglia incubated with epHero apoptotic cells for up to 24 h. **E** Percentage phagocytosing cells from amino acid-fed or -starved microglia co-cultured with epHero bait for up to 24 h. Dark grey: complete medium; light grey: without amino acids. **F** mCherry/eGFP ratio from amino acid-fed or starved microglia that have engulfed cargo after being co-cultured with apoptotic epHero bait for up to 24 h. **G** Representative histograms from **F**. **A**, **B**, **D**, **E**, **F** all data points depict mean ± SEM from *n* = 3 independent experiments (two-way ANOVA with Šídák’s multiple comparisons test).

To validate our epHero-based method for measuring efferocytosis, we serum-starved microglia (in the presence of amino acids) for 12 h before co-culturing them with epHero bait. Serum contains several pro-opsonisation molecules, including the complement component C1q, which facilitate the binding of apoptotic cells to phagocytes, an important first step in efferocytosis [29–31]. Serum starvation should therefore reduce the engulfment of apoptotic cargo by microglia, with minimal impact on its subsequent processing. As expected, serum starvation reduced the uptake of apoptotic cells by microglia (Fig. 6A). In serum-starved microglia that had engulfed apoptotic bait, phagosomal acidification was unaltered compared to their serum-fed counterparts (Fig. 6B, C). Importantly, this engulfment defect could be restored upon the re-addition of serum (Fig. 6D).

Recently, several groups have shown that amino acid metabolism affects apoptotic corpse clearance by macrophages [32–34]. To determine whether this also held true in microglia, HMC3 microglia were pre-treated in either complete or amino acid-deficient medium (in the presence of dialysed serum) for 12 h, and then co-cultured with apoptotic epHero bait. Amino acid starvation did not affect the uptake of apoptotic cargo (Fig. 6E). However, amino acid-starved microglia had markedly increased acidification of efferocytic material with time, as evidenced by the increased mCherry/eGFP ratio (Fig. 6F, G). Thus, the epHero tool is well-suited for capturing dynamic changes to the flux of efferocytic cargo.

## Discussion

The efficient removal of dead cells via efferocytosis is essential for maintaining homeostasis in an organism. Defects in this process have been implicated in a range of pathologies, from neurodegeneration [35] to cardiovascular disease [8] and autoimmune conditions [6, 7]. Here, we have harnessed differences in the fluorescent properties of mCherry and eGFP to generate a tandem-fluorescent epHero probe capable of tracking the acidification of the apoptotic corpse-containing phagosome. We show that epHero is a specific pH-sensitive reporter of efferocytosis flux in both microglia- and macrophage-like cell lines and is compatible with flow cytometry and imaging modalities – two of the most widely used phagocytosis readouts in the field. As proof-of-principle, we demonstrate that, in line with a previous report [31], the absence of serum reduces the uptake of apoptotic cells by phagocytes. Intriguingly, epHero revealed that amino acid starvation increases the acidification of apoptotic material without affecting its uptake – a phenomenon which should be explored further. Taken together, our data highlight the benefits of employing the epHero reporter to measure the simultaneous engulfment and acidification of apoptotic cells during efferocytosis.

Efferocytosis is a multi-step process, comprising an initial ‘find-me’ stage where phagocytes respond to factors released by apoptotic cells by moving towards the site of injury, an ‘eat-me’ stage where phagocytic receptors make initial contact with ligands expressed by apoptotic cells, and, finally, the internalisation and digestion of apoptotic cargo by the phagocyte [2]. However, to date, the efferocytosis field has largely focused on the uptake and engulfment of apoptotic material, with few studies examining post-engulfment processes. This has been driven, at least in part, by the paucity of readily available techniques to track the fate of the apoptotic corpse after phagocytic engulfment. At present, the most widely used assay to measure efferocytosis involves labelling apoptotic cargo with pH-responsive dyes such as pHrodo [10, 11, 27] and CypHer [5] which brightly fluoresce once the cargo has been taken up by phagocytes. Labelling apoptotic cargo with pH-responsive dyes cannot provide a sensitive measure of cargo acidification because an increase in signal could be driven by changes in either uptake or acidification. Further, quenching of the pHrodo or CypHer signal could indicate complete cargo digestion or defects in acidification. These differences can only be elucidated by combining these techniques with temporal and imaging data, making this a time- and labour-intensive undertaking. We addressed some of these limitations with our dual fluorescent probe, which enabled simultaneous measurement of both the uptake and acidification of apoptotic cargo. We compared our epHero probe to pHrodo, the current gold-standard in the field, and demonstrated that at early time-points, epHero performs similarly to pHrodo. However, 6 h post-engulfment there is a decrease in pHrodo signal compared to epHero. This is likely driven by lysosomal degradation of the pHrodo-protein conjugate, whereas mCherry remains stable within lysosomes [17]. If solely utilising the pHrodo probe, this decrease in both the proportion of pHrodo-containing cells and the total pHrodo signal could be misinterpreted as an overall decrease in efferocytosis rather than an increase in the acidification of phagosomal cargo. Thus, while previous studies have attempted to address the challenges of measuring efferocytosis, our epHero reporter provides distinct advantages over existing approaches.

To date, efferocytosis has been mostly measured in macrophages, with the results extrapolated to microglia [36]. This is not ideal, as microglia have a different lineage to tissue macrophages and operate in the brain’s unique environment [37]. In the present study, we tested our epHero probe in both macrophage and microglial cells and showed that it can be used to track corpse clearance of these two types of phagocytes. It has become apparent that phagocytic capacity differs in different cell types – for instance, efferocytosis in murine tissue-resident macrophages differs from that in peritoneal macrophages [38]. Future work using our epHero approach to systematically assay cargo acidification profiles in various phagocytes would provide an informative view on tissue-specific removal of apoptotic material.

In recent years, several groups have reported the importance of phagosomal acidification and cargo degradation during efferocytosis [28, 32], highlighting the need to successfully measure these processes. One study circumvented the limitations of conventional efferocytosis techniques by combining the pHrodo approach with GFP-expressing apoptotic cells, and employing the ratio of pHrodo to GFP fluorescence to measure acidification [28]. However, the signal of a fluorescent dye and an intracellular fluorophore can vary greatly. Given that our tandem fluorescent approach bypasses the need for an extra labelling step and ensures that mCherry and eGFP are expressed in equal amounts and present within the same cellular compartments, we consider that our reporter offers several improvements over this technique. Another group has developed an efferocytosis tool based on engineered red and green fluorophores and utilised it to measure efferocytosis in *Drosophila* [39], in an approach similar to our own. However, their probe is activated by caspase 3-induced cell death, which, while innovative, cannot be used to measure efferocytosis during caspase 3-independent programmed cell death [40, 41]. Additionally, our reporter relies on mCherry and eGFP fluorophores, which should be simple for most cell biology laboratories to repurpose for their own experiments. Further, we demonstrate that epHero can be used to detect changes to acidification during efferocytosis in mice.

The proposed new cost-effective methodology has several advantages over other techniques. epHero can dissect uptake from acidification of the corpse. This probe can also be used to measure efferocytosis of corpses that died by different pathways and is not limited to apoptosis. Further, we have demonstrated differences in phagosome acidification in a mouse model – this could be adapted for different disease models where cell death plays a prominent role, such as in neurodegenerative disease, stroke, and cardiovascular disease. Limitations to our technique include the necessity of generating genetically modified cells to use this approach. Another limitation is the fact that while quenching of eGFP does indicate an acidic environment, strictly speaking this may not result in degradation. Degradation can only be inferred, especially because mCherry is protease resistant in mammalian cells.

In summary, our epHero reporter provides efferocytosis researchers with a new tool to measure the uptake and acidification of apoptotic cargo concomitantly, both in vitro and in ex vivo models of efferocytosis. By expressing this probe downstream of a cell type-specific driver, future studies could build on work reported here to provide a true in vivo measure of murine efferocytosis. With its ability to track efferocytosis over time, the epHero reporter can be used to dissect the molecular mechanisms underlying dead cell clearance and their role in health and disease.

## Materials and methods

### Materials

Pharmacological reagents chloroquine (C6628; Sigma Aldrich St. Louis, Missouri, USA), bafilomycin (Sigma Aldrich SML1661) and cytochalasin D (Sigma Aldrich C2618) were used in this study. Commercial kits for pH calibration (P35379; Invitrogen Waltham, Massachusetts, USA), pHrodo labelling (Invitrogen P35358), Annexin V staining (640911 and 422201; Biolegend San Diego, California, USA), transfection (Invitrogen L000001) and western blot luminescence (34095 and 34580; Thermo Fisher Waltham, Massachusetts, USA) were employed. The following antibodies were used: anti-mCherry (ab167453; Abcam Cambridge, UK), anti-GFP (Invitrogen A6455), anti-LAMP1 (in-house [42] or Abcam ab24170), anti-β-actin-HRP (Sigma Aldrich A3854) and anti-F4/80 (40781l; Cell Signalling Technologies, Danvers, Massachusetts, USA).

### Animals

All animal experiments were approved by the South Australian Health and Medical Research Institute Animal Ethics Committee (SAM-20–075 or approved as a part of scavenging applications) and conducted in accordance with the *NHMRC Australian Code for the Care and Use of Animals for Scientific Purposes 2018*. Equal numbers of both male and female adult C57BL/6 mice were obtained from an established breeding colony and housed under standard conditions with a 12 h day/night cycle and *ad libitum* access to food and water.

### Cell culture

HMC3 microglial cells were cultured in EMEM containing 10% FCS. THP1 monocytes and Jurkat cells were maintained in RPMI 1640 medium supplemented with 10% FCS and 2 mM L-glutamine. THP1 monocytes were differentiated into macrophages via the addition of 80 nM PMA for 48 h. Following this, cells were allowed to recover in PMA-free medium for a further 24 h before being used in experiments. HEK293T cells were cultured in DMEM with 10% FCS. Bone marrow-derived macrophages were obtained by flushing mouse femurs and tibiae from 8–10 week old male mice C57/BL6 mice, and culturing bone marrow cells in RPMI containing 10% FCS, 1% Penicillin/Streptomycin and 50 ng/mL M-CSF. Bone-marrow cells were differentiated for 5 days before being harvested for experiments. All cell lines used in this study were maintained at 37 °C with 5% CO_2_, and routinely screened for mycoplasma infection.

For amino acid starvation experiments, HMC3 microglia were treated for 12 h in EBSS containing 10% dialysed FCS ± amino acids prior to performing the efferocytosis assay. For serum starvation experiments, HMC3 microglia were treated for 12 h in EBSS containing amino acids ±10% dialysed FCS prior to performing the efferocytosis assay. HMC3 microglia were either starved for the entire assay duration or re-stimulated with serum immediately prior to the addition of apoptotic bait.

### Generation of mCherry-eGFP expressing bait cells

#### Production of mCherry-eGFP lentiviral plasmid

The mCherry-eGFP coding sequence was PCR-amplified from Lenti-tf-APP [43] using the following primers: Forward 5’ – taagcaaccggtatgGTGAGCAAGGGCGAGGA and reverse 3’ – gtggccctgagaattcTTTACTTGTACAGCTCGTCCAT. The PCR amplicon was flanked by a 5’ AgeI and 3’ EcoRI site to facilitate restriction cloning into the pUltraHot vector (Addgene plasmid #24130, a gift from Malcolm Moore). Restriction digestion of the vector and PCR amplicon with AgeI and EcoRI enabled directional ligation of mCherry-eGFP into the pUltraHot backbone to create a pUltraHot-mCherry-eGFP plasmid.

#### Lentiviral transduction

HEK293T cells were plated at 1.5 ×10^5^ cells per well in two wells of a six-well plate and grown overnight. The following morning cells were transfected with 5 µg mCherry-eGFP-expressing lentivector, 4 µg psPAX2 (Addgene plasmid #12260, a gift from Didier Trono) and 4 µg pCMV-VSV-G (Addgene plasmid #8454, a gift from Bob Weinberg) using Lipofectamine 3000, according to the manufacturer’s instructions. The next day, THP1 or Jurkat target cells were seeded in two wells of a six-well plate at 1.5 ×10^5^ cells per well and maintained overnight. Two days post-transfection of HEK293T cells, culture medium containing mCherry-eGFP-expressing lentivirus was filtered using a 0.45 µM filter and polybrene added at a final concentration of 4 µg/mL. This medium was used to infect target cells overnight. The following day, virus-containing medium was replaced with complete growth medium, and transduced cells were maintained until flow cytometry analysis.

#### Generation of stable monoclonal cell lines

Lentivirus transduced cells were harvested, and mCherry-eGFP double-positive cells were sorted into 96-well plates at 1 cell per well on a BDAria Fusion cell sorter. Sorted cells were maintained in RPMI containing 20% FCS for up to 14 days for expansion into single colonies. Colonies were screened using fluorescence microscopy, and those with stable, moderate- to high expression of mCherry and eGFP were allowed to amplify further by sequentially transferring them to 24-, 12- and six-well plates. Clonal cell lines were analysed using flow cytometry on a BDFortessa, and those with high, consistent fluorescence peaks for both mCherry and eGFP with minimal variation were deemed the best clones and selected for all future experiments.

### Induction and verification of apoptosis in bait cells

Bait cells expressing mCherry-eGFP were rendered apoptotic via UV irradiation at 316 nm for 3–4 min, followed by incubation for 3 h at 37 °C with 5% CO_2_. Apoptosis was confirmed by Annexin V-Alexa Fluor® 647 staining according to the manufacturer’s instructions. Briefly, cells were pelleted and re-suspended in Annexin V binding buffer containing 0.25 µg/mL Annexin V-Alexa Fluor® 647 and 1 µg/mL DAPI and incubated in the dark for 15 min at room temperature before being washed with PBS. A live-dead cell cocktail was utilised to set the appropriate gating controls. All samples were analysed on a BDFortessa cytometer with >20,000 events recorded per condition.

### In vitro efferocytosis assay

HMC3 microglia, PMA-differentiated THP1 macrophages or bone marrow-derived macrophages were seeded in a 12-well plate at 1 ×10^5^ cells per well and maintained overnight. The following day, apoptotic bait cells were prepared as described above and added to the phagocytes at a 5:1 bait to phagocyte ratio for the indicated times. Phagocytes underwent three PBS washes to remove any unattached bait, were dissociated from the plate using trypsin to cleave un-internalised bait, and then analysed using flow cytometry. For pHrodo-based efferocytosis experiments, apoptotic bait cells were labelled with pHrodo Deep Red as per manufacturer’s instructions. Where indicated, phagocytes were pre-treated with 10 µM cytochalasin D, 100 nM bafilomycin or 50 µM chloroquine for 10 min before the addition of bait cells.

### Ex vivo efferocytosis measurements

Apoptotic epHero bait cells were prepared as described above. Mice were intraperitoneally injected with either chloroquine (50 mg/kg) or saline, as well as 4 ×10^5^ apoptotic cells re-suspended in 200 µL of HBSS. Mice injected with either no cells or non-fluorescent apoptotic THP1 cells served as the control. At 1- or 4 h post-injection, mice were humanely killed and peritoneal cavity lavage was performed. Recovered cells were pelleted, resuspended in FACS buffer and stained with F4/80 (1:40) for 30 min on ice. Cells were then washed and resuspended in FACS buffer. Flow cytometry measurements were performed on a BDFortessa with gating based on unstained cells and mice not injected with apoptotic epHero bait. Both male and female mice were used in this study. All flow cytometry data were analysed on FlowJo™ version 10.8 (BD Life Sciences, Franklin Lakes, New Jersey, USA).

### Western blotting

THP1 or epHero THP1 cells were rendered apoptotic as described above, pelleted, and resuspended in cell lysis buffer containing protease and phosphatase inhibitors. Samples were sonicated on ice, and protein concentration determined via a BCA assay. Following this, 10 µg protein was loaded per well and electrophoresed through 4–12% SDS-PAGE gels before being transferred to a PVDF membrane as previously described [41]. The membranes were blocked in a solution comprising Tris-buffered saline containing 0.1% (v/v) Tween 20 and 5% (w/v) skim milk and incubated overnight at 4 °C with anti-mCherry (1:2 000) and anti-eGFP (1:1 000) antibodies. Incubation with HRP-conjugated anti-mouse or anti-rabbit secondary antibodies (1:10 000) was performed at room temperature for 1 h. To detect β-actin, membranes were incubated with an HRP-conjugated anti-β-actin antibody (1:10 000) for 1 h at room temperature. Membranes were developed on the LAS4000 Luminescent Image Analyser (Fujifilm Life Science, Cambridge, Massachusetts, USA) using the West Pico or West Femto ECL systems.

### Confocal microscopy

Bait cells were seeded into 12-well plates containing poly-L-lysine-coated coverslips at 1 ×10^5^ cells per well and grown overnight. The following day, cells were either rendered apoptotic as described above, or left as untreated controls. Non-adherent cells were aspirated, and coverslips fixed with 10% formalin for 10 min. Coverslips were washed twice with PBS, mounted onto slides using Vectashield Hardset with DAPI, and sealed with nail polish.

For live imaging, HMC3 cells were seeded into two-well chambered coverslips at 1 ×10^5^ cells per well. The following day, epHero THP1 cells were rendered apoptotic and added to HMC3 cells at a 5:1 bait to phagocyte ratio. Live imaging was performed in a humidified chamber at 37 °C and 5% CO_2_, with z-stacks (4–5 optical sections, z range ~3 µm) taken every 10 min for 2 h.

For immunostaining, HMC3 microglia or bone-marrow derived macrophages were seeded onto poly-L-lysine coated coverslips at 1.5 ×10^5^ cells per well and left to grow overnight. Apoptotic THP1 or Jurkat epHero bait cells were co-cultured with phagocytes for 2 h at a 5:1 bait to phagocyte ratio. Phagocytes were washed three times with PBS to remove un-internalised bait, and fixed with 10% formalin for 10 min. For some experiments, cells were permeabilized in a buffer of PBS with 0.1% saponin. Cells were blocked in PBS with 5% BSA and 0.01% saponin buffer, and then stained with anti-GFP (1:100), anti-mCherry (1:250) or anti-Lamp1 (1:250) primary antibodies for 1 h at room temperature. Coverslips were washed with PBS and incubated with secondary antibodies (1:500) for 1 h at room temperature, washed and mounted onto slides using Vectashield Hardset with DAPI.

All images were acquired using a TCS SP8X confocal microscope with LASX (Leica, Germany) and imported into ImageJ for analysis.

### Data analysis

FlowJo™ version 10.8 (BD Life Sciences) was used to analyse all flow cytometry data. All imaging data were compiled for publication in ImageJ version 2.14. All statistical analysis was performed using GraphPad version 10. Sample size and statistical tests for each experiment are detailed in the Figure legend. In general, data were analysed via either t-test, one-way or two-way ANOVA. Gaussian distribution was verified using a Shapiro-Wilk test, and either parametric or non-parametric analyses were performed accordingly. Data are presented as either individual data points or means with standard error bars as indicated in the Figure legends. *p* ≤ 0.05 was considered statistically significant.

### Supplementary information

Original data

Supplementary figures

Supplementary Video 1

## Data Availability

The datasets generated during the current study are available from the corresponding author on reasonable request.
