# Supplementary figures and images for: epHero – a tandem-fluorescent probe to track the fate of apoptotic cells during efferocytosis
Source: Cell Death Discov. 2024 Apr 17;10:179. doi: 10.1038/s41420-024-01952-1 (PMC11024195; doi:10.1038/s41420-024-01952-1)

**
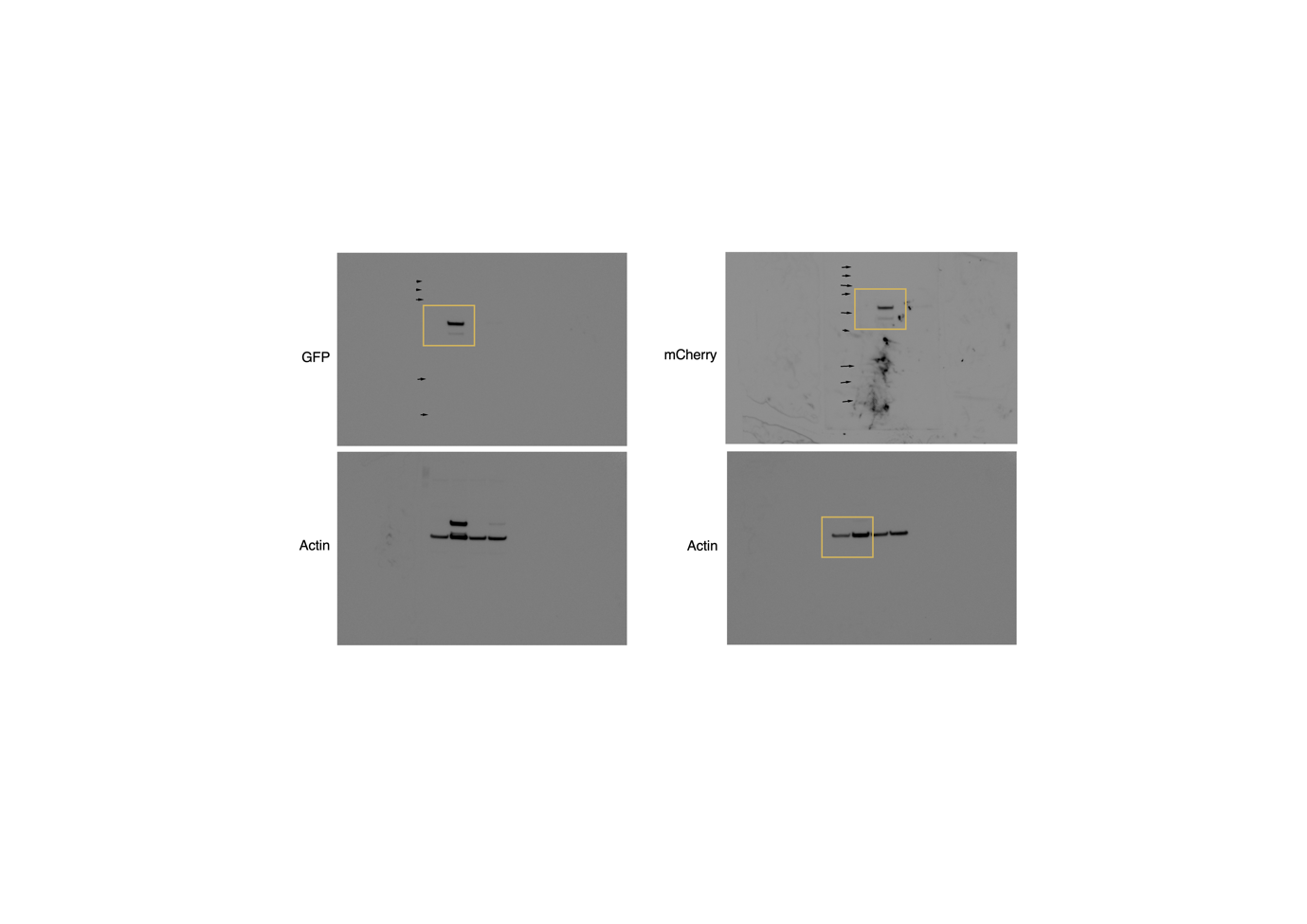
 Original data.** Uncropped western blots used to generate Figure 1D.

Supplement: Supplementary file 1 — Original data [file 41420_2024_1952_MOESM1_ESM.docx]
