# Supplementary material for: epHero – a tandem-fluorescent probe to track the fate of apoptotic cells during efferocytosis
Source: Cell Death Discov. 2024 Apr 17;10:179. doi: 10.1038/s41420-024-01952-1 (PMC11024195; doi:10.1038/s41420-024-01952-1)
Supplement: Supplementary file 2 — Supplementary figures [file 41420_2024_1952_MOESM2_ESM.docx]

**
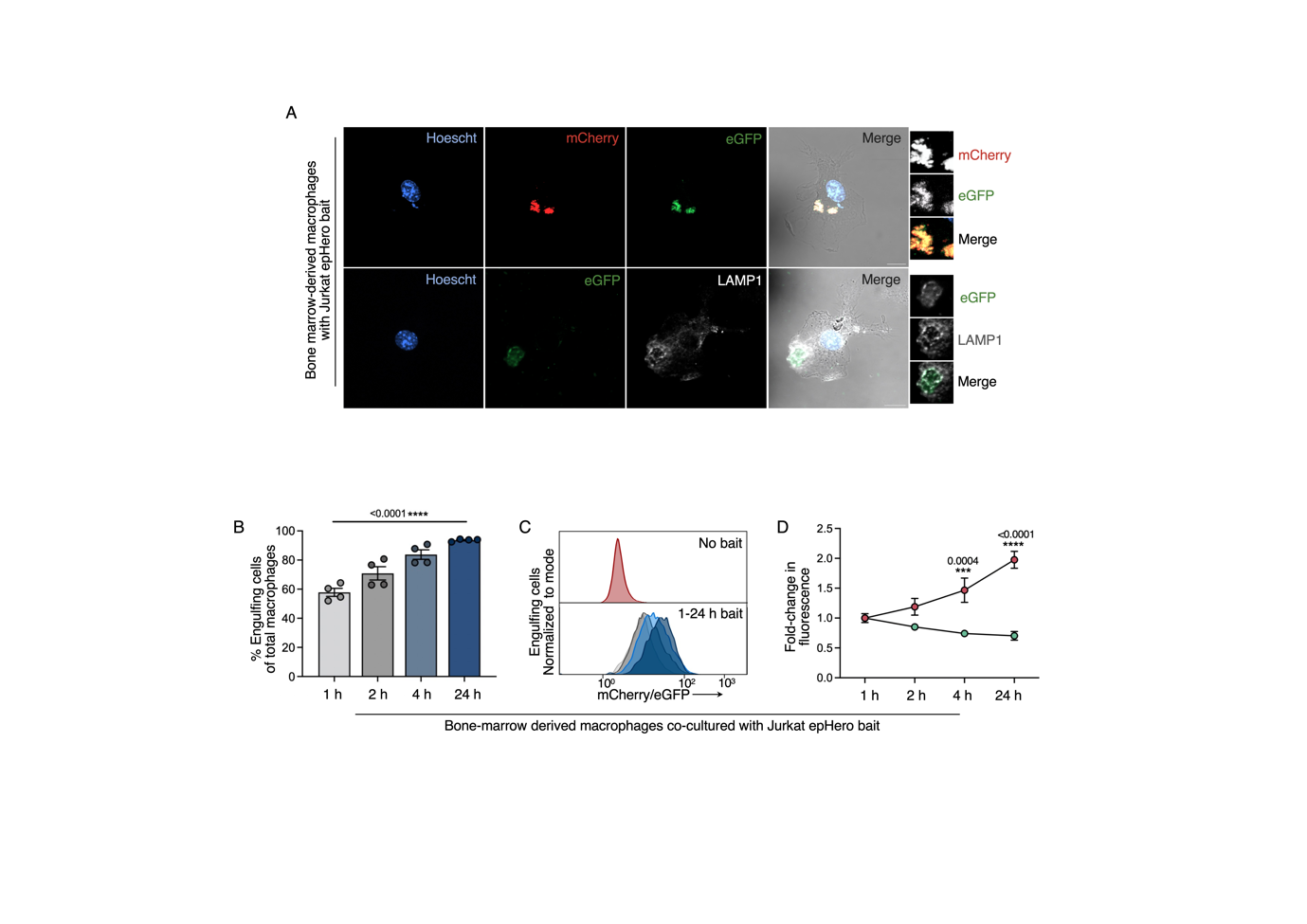
Supplementary Figure 1. Jurkat epHero bait taken up by bone-marrow derived macrophages. (A)** Representative images of bone marrow-derived macrophages co-cultured with apoptotic Jurkat epHero cells for 2 h and either probed for mCherry and eGFP fluorescence (panel 1) or immunostained with an eGFP and LAMP1 antibody (panel 2). Scale bars: 10 µM. **(B)** Percentage of phagocytosing cells from macrophages co-cultured with apoptotic epHero bait for up to 24 h. Data points centred on mean ± SEM with n = 4 from two independent experiments (one-way ANOVA with test for linear left-to-right trend). **(C)** Representative flow cytometry histograms from *(B)*. **(D)** Fold-change in fluorescent signal (relative to 1 h) over time in phagocytosing macrophages from *(B).* Red: mCherry fluorescence; Green: eGFP fluorescence. Data points denote mean ± SEM (two-way ANOVA with Šídák’s multiple comparisons test).

**
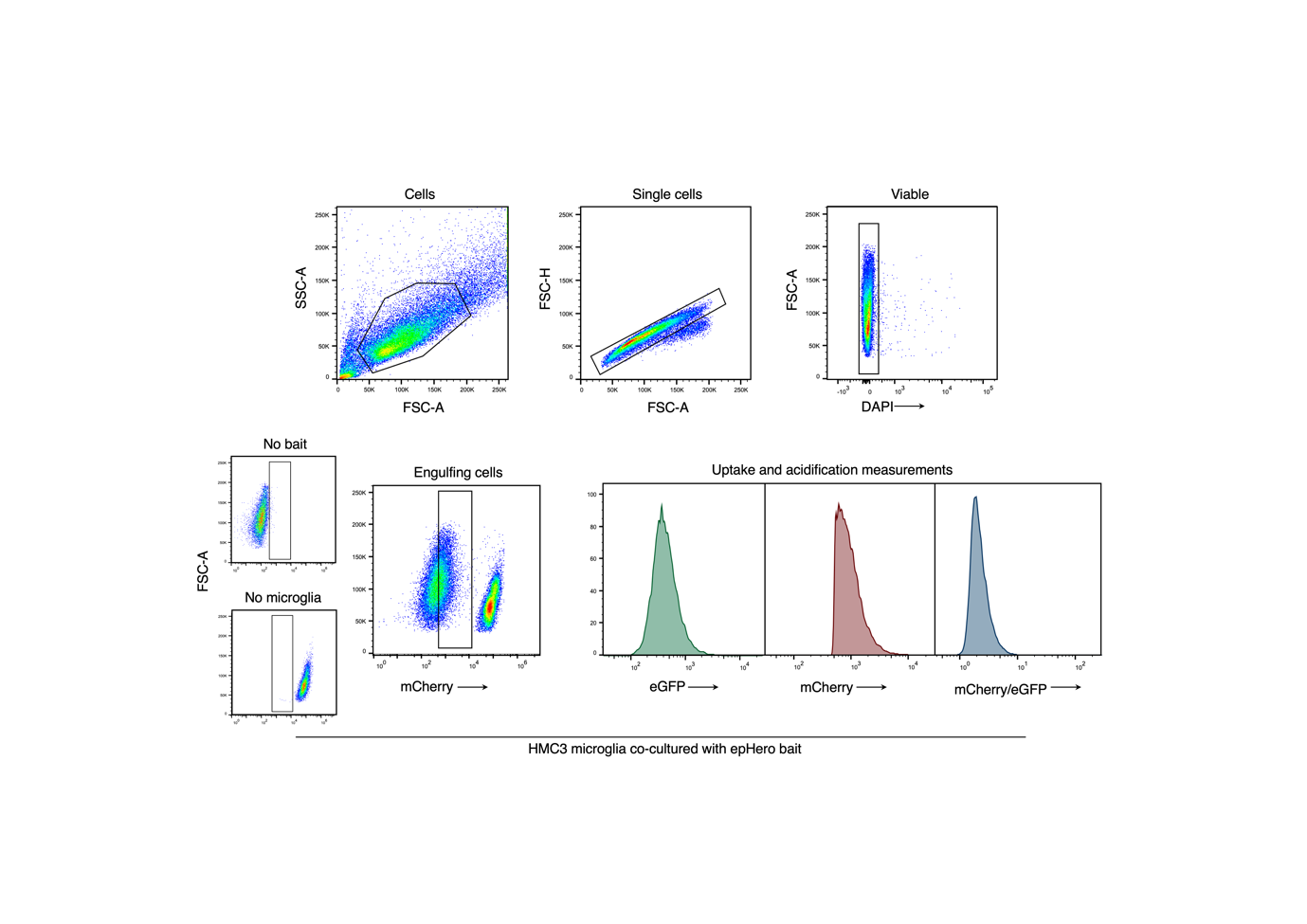
Supplementary Figure 2. Flow cytometry gating strategy.** Gating used for *in vitro* efferocytosis experiments.

**
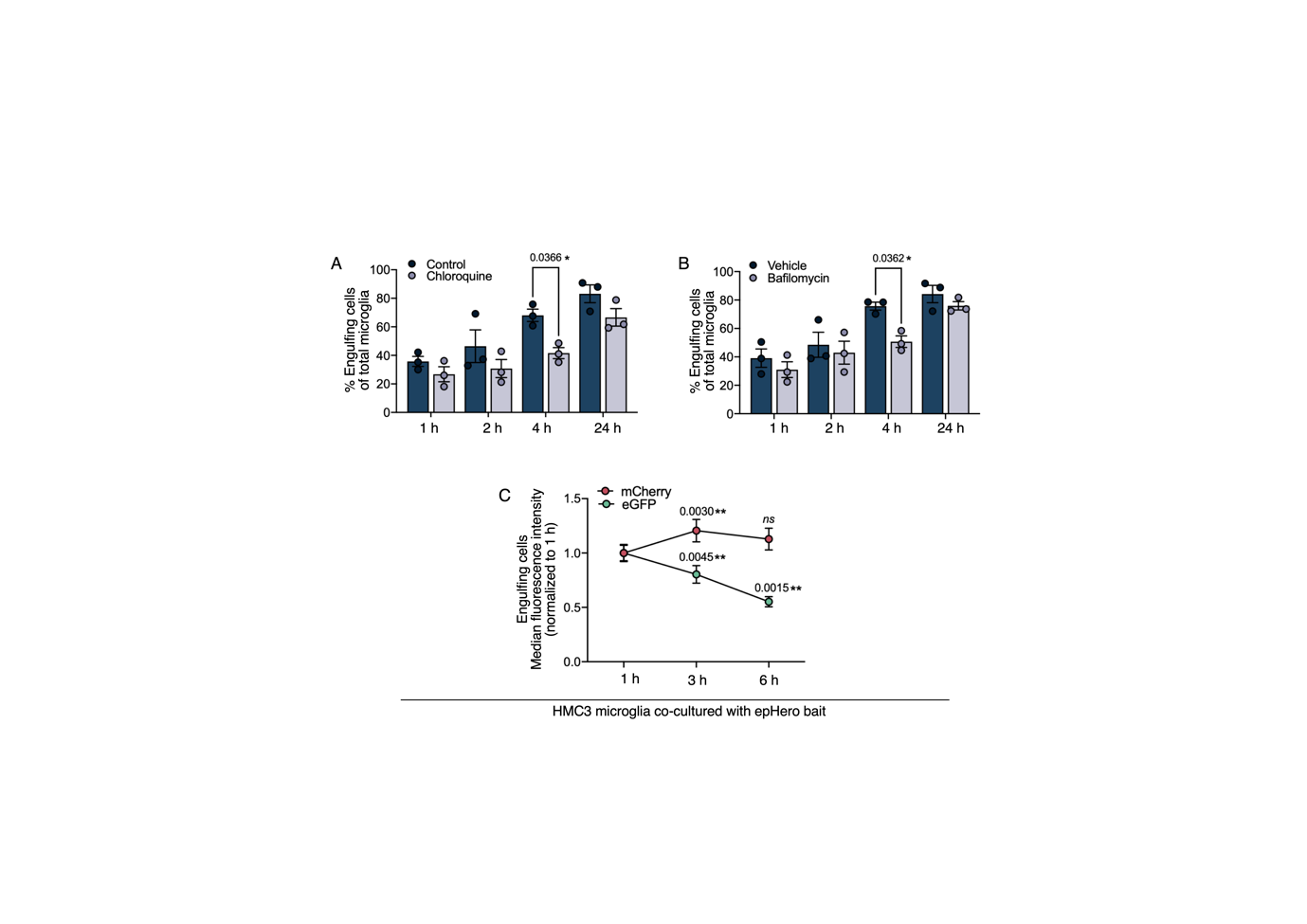
Supplementary Figure 3. pH sensitivity of the epHero reporter when used to track efferocytosis. (A)** Percentage of microglia that have phagocytosed cargo after being co-cultured with apoptotic epHero bait for 1-, 2-, 4- or 24 h, with or without 50 µM chloroquine. **(B)** Percentage of microglia engulfing cargo when co-cultured with apoptotic epHero bait for 1-, 2-, 4- or 24 h, with or without 100 nM bafilomycin. *(A), (B)* Data represent mean ± SEM from n = 3 independent experiments (two-way ANOVA with Šídák’s multiple comparisons test). **(C)** Relative mCherry and eGFP fluorescence in microglia engulfing epHero cargo from Figure 1G. Data represent mean ± SEM from n = 6-8 across three- to four independent experiments (two-way ANOVA with Šídák’s multiple comparisons test).

**Supplementary Figure 4. Efferocytosis in the mouse peritoneal cavity. (A)** Flow cytometry gating strategy for mouse experiments in Figure 5. **(B)** Percentage of mouse peritoneal F4/80+ macrophages that have phagocytosed cargo, from control (closed circles) or chloroquine-treated (open circles) mice 1- to 4 h following injection with apoptotic epHero bait. Data points are centred on mean ± SEM, with 7-9 mice per condition (two-way ANOVA with Šídák’s multiple comparisons test).

**Supplementary Video 1. Video depicting an epHero cargo acidification event in a HMC3 microglial cell.** Stills from this video are presented in Figure 4A.
